# Transgenic miR132 Alters Neuronal Spine Density and Impairs Novel Object Recognition Memory

**DOI:** 10.1371/journal.pone.0015497

**Published:** 2010-11-29

**Authors:** Katelin F. Hansen, Kensuke Sakamoto, Gary A. Wayman, Soren Impey, Karl Obrietan

**Affiliations:** 1 Department of Neuroscience, Ohio State University, Columbus, Ohio, United States of America; 2 Department of Veterinary and Comparative Anatomy, Pharmacology and Physiology, Washington State University, Pullman, Washington, United States of America; 3 Oregon Stem Cell Center, Oregon Health and Science University, Portland, Oregon, United States of America; Johns Hopkins, United States of America.

## Abstract

Inducible gene expression plays a central role in neuronal plasticity, learning, and memory, and dysfunction of the underlying molecular events can lead to severe neuronal disorders. In addition to coding transcripts (mRNAs), non-coding microRNAs (miRNAs) appear to play a role in these processes. For instance, the CREB-regulated miRNA miR132 has been shown to affect neuronal structure in an activity-dependent manner, yet the details of its physiological effects and the behavioral consequences *in vivo* remain unclear. To examine these questions, we employed a transgenic mouse strain that expresses miR132 in forebrain neurons. Morphometric analysis of hippocampal neurons revealed that transgenic miR132 triggers a marked increase in dendritic spine density. Additionally, miR132 transgenic mice exhibited a decrease in the expression of MeCP2, a protein implicated in Rett Syndrome and other disorders of mental retardation. Consistent with these findings, miR132 transgenic mice displayed significant deficits in novel object recognition. Together, these data support a role for miR132 as a regulator of neuronal structure and function, and raise the possibility that dysregulation of miR132 could contribute to an array of cognitive disorders.

## Introduction

microRNAs (miRNAs) are small, evolutionarily conserved, molecules that act as potent silencers of gene expression via translational repression and/or mRNA destabilization [1]. Since their characterization in C. elegans [2], [3], there has been an explosion of studies revealing fundamental and critical roles for miRNAs in virtually all aspects of cell biology.

Within the nervous system, a good number of miRNAs exhibit developmentally-dependent and cell-type-specific, expression patterns [4]–[7]. Further, recent work has shown that miRNAs can be expressed in an activity-dependent manner [8], [9]. Within this context, particular attention has been paid to the miRNA132 (miR132). miR132 is processed from the intron of a small non-coding RNA gene and is robustly responsive to an array of physiological and pathophysiological stimuli [10]–[16]. With respect to neuronal function, miR132 has been shown to influence dendritic growth and spinogenesis in cultured hippocampal neurons and in brain slices [10], [12], [17]. Some of these effects appear to be mediated by the down regulation of the miR132 target p250GAP, which, in turn, allows for Rac1-PAK-mediated spinogenesis [12], [13]. Interestingly, expression of methyl CpG–binding protein 2 (MeCP2) is also tightly regulated by miR132 [18], and altered expression of MeCP2 has been shown to be an underlying element in the development of Rett syndrome, a neuro-developmental disorder in which dendritic development and synaptogenesis are affected [19]–[21]. Thus, miR132 appears to be well-positioned to couple synaptic activity to neuronal structural/functional plasticity.

To begin to address the potential role of miR132 *in vivo*, we developed a transgenic mouse strain that over-expresses miR132 in forebrain neurons. Morphometric analysis revealed a marked effect of transgenic miR132 on hippocampal neuronal morphology. Concordant with this, there was a decrease in MeCP2 expression in hippocampus. Furthermore, we show that these mice have impaired hippocampal-dependent object recognition memory. Collectively, these data reveal miR132 as a potent regulator of neuronal structure and CNS function.

## Materials and Methods

### Ethics Statement

All animal breeding and experimental procedures were approved by the Ohio State University Animal Care and Use Committee (protocol number: 2008A0227).

### Generation of miR132 transgenic mice

The tetracycline regulated bi-directional pBI-G vector (Clontech, Mountain View CA) was modified by removing the lacZ coding region, and replacing it with enhanced cyan fluorescent protein (CFP: subcloned from the pCX-ECFP vector). To shorten the half-live of CFP, and thus allow for a more dynamic read-out of transgene expression, the mouse ornithine decarboxylase PEST sequence was subcloned, in frame, to the 5′ end of CFP. The premiR132 sequence from the pCAG-miR132 vector [10] was subcloned into the multiple cloning site of the pBI-CFP vector to generate the final TET-response element-controlled miR132-CFP vector. Dr. Hai-Ying M. Cheng performed the subcloning procedures: pronuclear injection of FVB/N oocytes with the linearized construct was performed Dr. Xin-An Pu, at the Ohio State University Transgenic Facility. Five lines were generated and analyzed for transgene expression by crossing to the CaMKII tTA driver line [22]. The line with the broadest neuroanatomical expression pattern was retained and backcrossed to the C57BL/6J mouse strain a minimum of 6 times. Of note, at no point were the animals administered with doxycycline. Thus the miR132 transgene was constitutively expressed. Thy-1 GFP mice [23] were generously provided by Gouping Feng (Duke University).

### RT-PCR miRNA quantitation

Total hippocampal and cortical RNA was isolated, and reverse-transcribed using the miScript Reverse Transcription Kit (Qiagen, Valencia, CA USA). miRNAs were polyadenylated with Poly(A)polymerase before reverse transcribing with universally-tagged 5′ oligo-dT primer (Qiagen). cDNA was then amplified and miR132 was quantified by real-time PCR analysis using the miScript Primer System (Qiagen). Data were normalized to RNU6B_2 cDNA levels.

### Tissue Processing and Immunolabeling

Brain tissue was removed after cervical dislocation and postfixed in 4% paraformaldehyde for 4 h at 4°C and cryoprotected with 30% sucrose in PBS. Brains sections (500 µm thick) were prepared using a vibratome and hippocampus-containing sections were then thin-cut (40 µm or 80 µm think) on a freezing microtome.

For immunohistochemical labeling, sections were washed in PBS and blocked with 10% normal goat serum in PBS, and then treated with 0.3% hydrogen peroxide. Sections were then incubated overnight (4°C) with rabbit polyclonal anti-MeCP2 antibody (1∶3000 dilution; Cell Signaling, Davners, MA). Next, the tissue was incubated for 2 h at room temperature in biotinylated anti-rabbit IgG (1∶300; Vector Laboratories) and then placed in an avidin/biotin HRP complex (Vector Laboratories, Burlingame, CA) for 1 h, following the manufacturer's instructions. The signal was visualized using nickel-intensified DAB substrate (Vector Laboratories), and sections were mounted on gelatin-coated slides with Permount media (Fisher Scientific, Petaluma, CA). Sections were washed in PBS (three times, 10 min per wash) between each labeling step.

Sections used for immunofluorescent labeling were washed and permeabilized in PBS with 1% Triton X-100 PBST for a total of 30 min. Sections were then blocked for 1 h in10% normal goat serum in PBS and incubated (overnight, 4°C) in mouse anti-NeuN antibody (1∶3000; Millipore, Billerica, MA) and/or rabbit polyclonal anti-GFP antibody (acquired from Dr. Luc G. Berthiaume, University of Alberta, Canada). The GFP antibody was used at 1∶2,500 to detect the TRE-regulated CFP transgene and 1∶20,000 to detect the thy1-regulated GFP transgene.

Next, sections were incubated (3 h at room temperature) in Alexa Fluor-594-conjugated goat anti-rabbit IgG antibody (1∶1000; Invitrogen, Carlsbad, CA) and/or Alexa Fluor-488-conjugated goat anti-mouse IgG antibody (1∶1000; Invitrogen). Tissues were washed in PBST (three times, 10 min per wash) between each labeling step. Sections were mounted on slides with Flouro-Gel (Electron Microscopy Sciences, Hatfield, PA).

### Analysis

Morphological changes in CA1 basal dendrites (ten per animal) were examined over two 40 µm-thick coronal sections per animal. Confocal images of Thy-1 GFP immunofluorescence were captured 90–120 µm from the cell soma and spines were examined over 20 µm dendritic segments. Processes that extended >0.5 microns from the dendrite were counted. Of note, processes that had a mushroom-shaped morphology and from those with a filopodia-like morphology (i.e., long, thin, protrusions) were counted and are referred to here as ‘spines’. Images were acquired at 63× with 4.3× optical zoom, using a Zeiss 510 Meta confocal microscope and LSM Software Zen.

MeCP2 labeling images were acquired at 10× using a 16 bit digital camera (Micromax YHS 1300; Princeton Instruments) mounted on an inverted Leica microscope (DM IRB). Images of the granule cell layer (GCL) and CA1 of MeCP2 labeled sections were traced digitally and intensity levels were analyzed with MetaMorph software (Molecular Devices). Intensity levels were normalized by background subtraction and presented relative to nontransgenic CA1 staining.

### Western Blotting

Isolated hippocampal tissue was lysed in 100 µl radioimmunoprecipitation assay buffer (50 mM Tris-HCl, 150 mM NaCl, 1 mM EDTA, 1% Triton X-100, 0.1% sodium dodecyl sulfate, 1% sodium deoxycholate, 1 mM sodium vanadate, 1 mM NaF, and 1× protease inhibitor cocktail (Roche). Protein extracts (5 µg/lane) were electrophoresed into a 10%SDS-PAGE gel and then transblotted onto polyvinylidene difluoride membranes (Immobilon-P; Millipore). Membranes were blocked in 5% milk in PBST and then incubated (overnight, 4°C) in PBST (with 5% BSA) with the MeCP2 antibody (1∶6000; Cell Signaling). Next, membranes were incubated in PBST (with 5% milk) with an anti-goat IgG horseradish peroxidase-conjugated antibody (1∶2000; Jackson Laboratories). As a protein loading control, membranes were probed for ERK1 expression using a goat polyclonal anti-ERK1 antibody (1∶1000; Santa Cruz Biotechnology) followed by anti-goat IgG antibody conjugated to horseradish peroxidase (1∶2000; PerkinElmer Life Sciences). The signal was visualized using the Western Lighting Chemiluminescence light-emitting system (PerkinElmer Life Sciences) and HyBlot CL autoradiography film (Denville Scientific). Between each antibody treatment, membranes were washed a minimum of four times (10 min per wash) in PBST. Densitometric band analysis was performed using Phototshop (Adobe Systems). For each lane, MeCP2 and ERK1 band intensity was background subtracted, and the MeCP2 signal was divided by the ERK1 signal; mean data for each group were derived from three animals (biological replicates) per condition.

### Behavioral Analysis

Novel object recognition was adapted from procedure described by Bevins and Besheer [24]. Each animal was allowed a 10 min training session with exposure to two identical, non-toxic objects (glass or hard plastic items) placed in the back left and right corners of the arena. After the training session, the animal was returned to its home cage for a 30-min retention interval. For testing, each animal was lowered into to the testing arena in which one familiar object was replaced with a novel object. The animal was lowered into the arena, equidistant and facing away from each object. Each session was video record and the animal was given 5 min to explore. The time spent exploring each object was scored for each mouse from the video. Exploration was defined as the animal's nose being within 2 cm of and pointed toward the object. Time during which the animal propped itself up on the object in order to explore higher levels of the arena was not considered exploration time for that object. The discrimination ratio was calculated as the time spent with the novel object divided by the total time spent exploring either object. Objects were randomized and counterbalanced across animals and groups. Objects and arenas were thoroughly cleaned with 70% ethanol between trials to prevent olfactory cues.

Of note, the visual ability of each mouse was assessed by suspending the animal by the tail and slowly lowering it toward a sold dark surface (a table) for three successive trials. Visual acuity was demonstrated by the animal's reaching for the surface before vibrissae made contact with it. All mice demonstrated full visual capability.

### Statistics

All values in the paper are given as means ±SEM and comparisons between groups were made by Student's t-test with significance set as p<0.05 unless otherwise noted. Significance for novel object recognition was assessed using one-way ANOVA analysis followed by Fisher's least significant difference (LSD) test. A value of p<0.05 was accepted as statistically significant.

## Results

### Bitransgenic expression of tTA::miR132 *in vivo*

**Figure 1:**
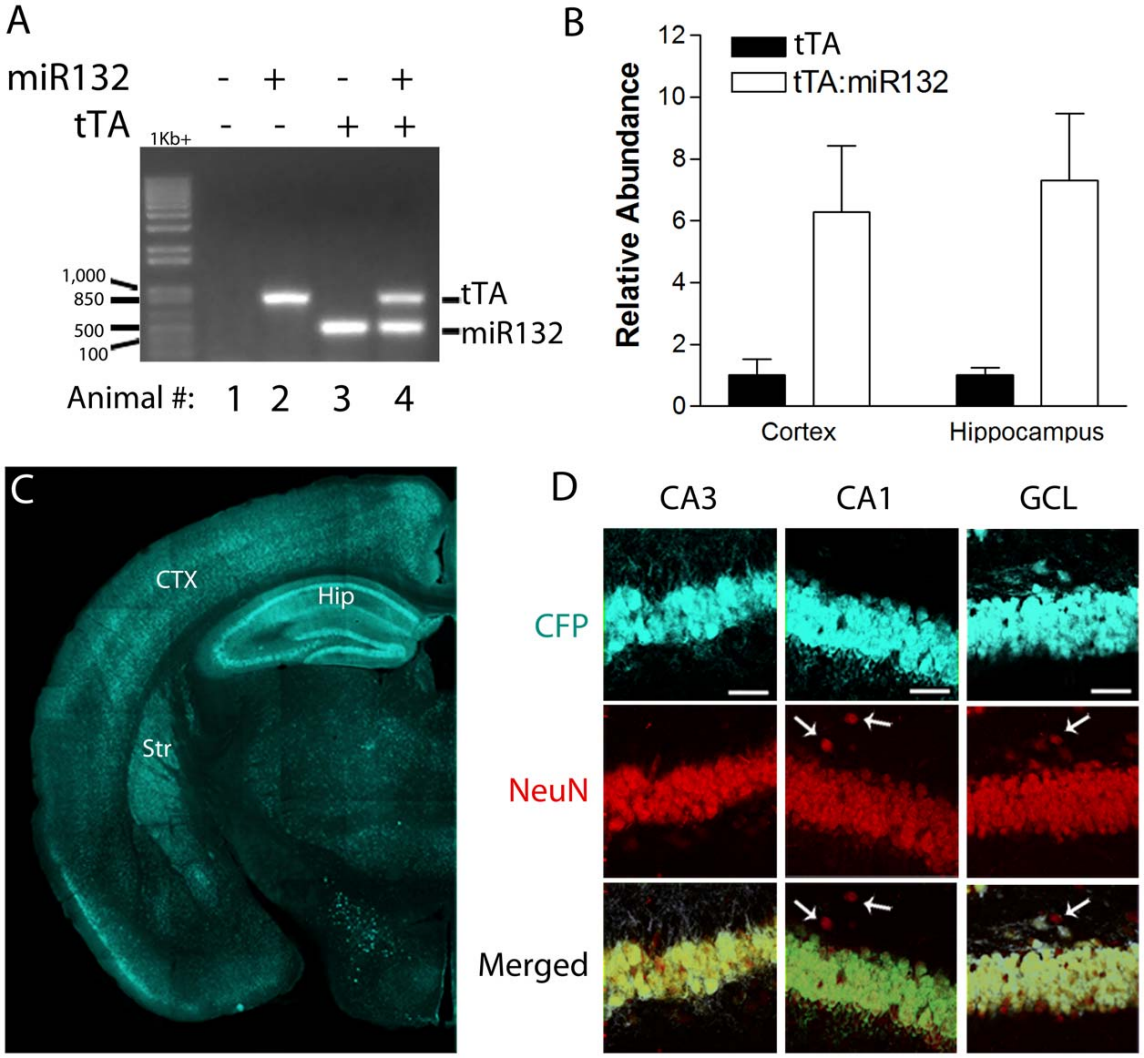
miR132/CFP transgene expression. (A) PCR-based genotyping results for the tTA and miR132; of note, only mouse #4 contains both the tTA and miR132 transgenes. PCR reactions were run in a 1% agarose gel and visualized using ethidium bromide. (B) To confirm miR132 overexpression in tTA::miR132 mice, total hippocampal and cortical RNA was isolated, reverse-transcribed, and the miR132 cDNA was profiled via real-time PCR. Normalized mature miR132 levels are shown as mean ±SEM, compared to nontransgenic controls (NT: n = 3 animals per condition). Data were normalized to RNU6B_2 cDNA levels. Representative transgene expression *in vivo*. (C) Coronal brains sections were immunolabeled for the CFP transgene marker. Note the robust transgene expression within the cortex (CTX), hippocampus (Hip) and striatum (Str). (D) Double immunofluorescent labeling for CFP and NeuN reveals that hippocampal excitatory neurons of the GCL and the CA1 and CA3 sublayers express the transgene. Arrows denote the location of nontransgenic neurons adjacent to the excitatory cell layers. Scale bars: 50 µm.

To explore the functional role of miR132 *in vivo*, our lab generated a tetracycline response transgenic mouse strain, which bidirectionally drives the expression of miR132 and cyan fluorescent protein (CFP). To target transgene expression to the forebrain neurons, these mice were crossed with mice expressing tTA via the CaMKII promoter [22] (Fig. 1A, B). Quantitative real-time RT-PCR revealed that the transgene lead to an ∼7-fold increase in the mature form miR132, relative to tTA monotransgenic littermates (Fig. 1B). Immunolabeling for CFP detected the expected expression pattern within forebrain regions, including the hippocampus and cortex (Fig. 1C). Further, double immunofluorescence labeling for CFP and the neuronal nuclear antigen (NeuN) revealed that cells with the pyramidal morphology, indicative of glutamatergic neurons, robustly expressed the transgene (Fig. 1D).

### Expression and morphology of Thy1-GFP positive neurons

**Figure 2:**
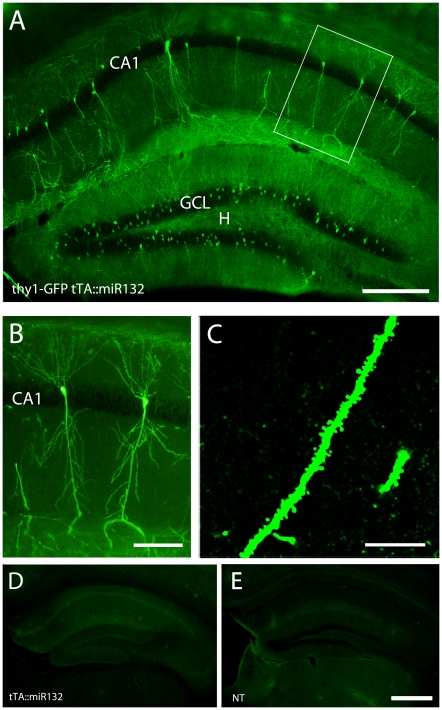
Hippocampal expression of Thy1-GFP. (A) Representative GFP fluorescent immunolabeling of the dorsal hippocampus. A limited subset of CA1 pyramidal neurons and granule cells express the GFP transgene. GCL, granule cell layer; CA1, hippocampal subfield; H, hilus. Framed CA1 pyramidal cell is shown at higher magnification (B), as well as a confocal image of a CA1 dendrite (C). (D) Immunolabeling for TRE-regulated CFP expression in a tTA::miR132 transgenic mouse. At the antibody concentration used to reveal Thy-1 driven GFP expression (presented in *A*), minimal expression of CFP was detected. (E) As a further control, wild type (WT) tissue was immunolabeled using the GFP antibody: minimal non-specific labeling was detected. Of note, all images presented here (except for the confocal image in C) used identical data collection and analysis settings. Scale bars: 200 µm in A, 100 µm in B, 10 µm in C, 200 µm in D.

Given prior work showing that miR132 affects the morphology of cultured neurons, we initially examined neuronal ultrastructure in tTA::miR132 mice. To this end, the tTA::miR132 mice were crossed with a transgenic line expressing green fluorescent protein (GFP) under the control of the *thy1* promoter. Thy-1 is expressed by projection neurons throughout many areas of the nervous system [25] and its promoter is used to transgenically drive robust GFP expression in a subpopulation of hippocampal neurons [26]. Importantly, Thy1-driven morphological marker does not affect the electrophysiological or the morphological properties (i.e., dendrite length and number, spine number and density, soma size) of hippocampal neurons [26]. Of note, for all morphometric studies, we employed a fluorescent immunolabeling approach, which increased (relative to native GFP fluorescence) our ability to detect the transgene. Notably, the expression level of the tet-responsive CFP transgene (which is also antigenic to the GFP antibody) was markedly lower than the Thy-1 driven GFP, and thus, we were able to selectively visualize the Thy-1 GFP transgene by using a relatively low concentration (1∶20,000) of the primary antibody. In support of this approach, immunofluorescence labeling of tTA::miR132 tissue with this antibody concentration did not allow for clear visualization of the CFP reporter (Fig. 2D). As a relative comparison, wild-type tissue was also labeled using the GFP antibody (Fig. 2E). Further, quantitative analysis of the immunofluorescence signal intensity did not detect a significant additive effect of Thy-1 GFP and miR132-CFP reporters, relative to Thy-1 GFP labeling alone (data not shown).

### Changes in dendritic morphology of tTA::miR132 mice

**Figure 3:**
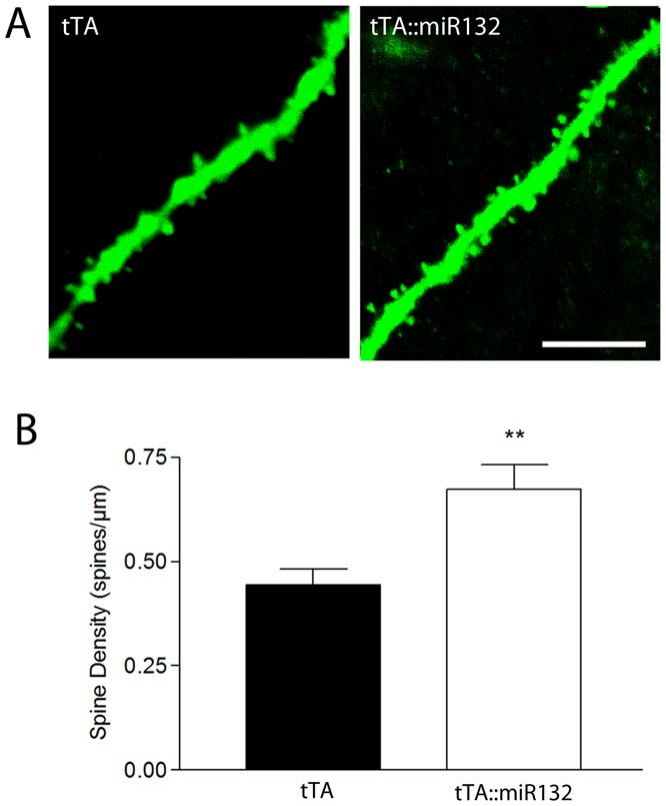
Transgenic miR132 affects neuronal morphology. (A) Representative confocal images of CA1 pyramidal neuron basal dendrites from tTA::miR132 transgenic and tTA monotransgenic tissue. Note the increased spine density in the tTA::miR132 dendrite compared the tTA transgenic mouse. (B) Graphical representation of the mean ± SEM spine density. **P<0.01, two-tailed t-test, n = 6 animals for each group. Please see the Methods section for a description of the quantification methods. Scale bar: 10 µm.

The spine density of CA1 neurons expressing the Thy1-GFP hippocampal neurons was measured from coronal sections over 20 µm lengths of basal dendrites from Thy-1::tTA::miR132 triple transgenic mice and from Thy-1::tTA: control littermates (Fig. 3A, B). Consistent with previous *in vitro* data on miR132, mice that express transgenic miR132 showed a significant increase in spine density in CA1 neuronal dendrites over Thy-1::tTA: control littermates. These data indicate that miR132 modulates neuronal structural features associated with synaptic communication.

### Decreases in MeCP2 levels in the tTA::miR132 hippocampus

**Figure 4:**
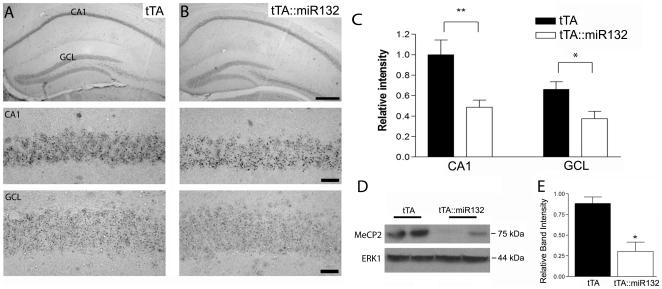
Decreased MeCP2 expression in tTA::miR132 mice. A and B: Top panels) Representative immunohistochemical labeling for MeCP2 in the dorsal hippocampus of tTA monotransgenic (A) and tTA::miR132 bitransgenic (B) mice. Relative to tTA littermates, mice over-expressing miR132 showed a decrease in MeCP2 expression within the excitatory cell layers of the hippocampus. Scale bar: 200 µm. Middle and bottom panels) High magnification images of MeCP2 labeling within area CA1 and the GCL are presented. Scale bar: 100 µm. (C) Quantitative analysis of the mean ± SEM MeCP2 expression in area CA1 and the GCL. Of note, MeCP2 expression was significantly reduced in tTA::miR132 mice (n = 7) compared to monotransgenic controls (n = 8) (**P<0.01, *P<0.05). (D) Western analysis of biological replicates (two animals per condition) confirming the reduction in MeCP2 expression in tTA::miR132 mice, along with quantitative densitometric analysis relative to endogenous ERK1 levels (E), presented as mean ± SEM (n = 3) (*P<0.05).

Given recent work using *in vitro* model systems showing that MeCP2 is a target of miR132 [18], we investigated whether transgenic miR132 affected MeCP2 levels in the hippocampus. Immunohistochemical labeling revealed significant decreases in MeCP2 expression in both the CA1 cell layer and GCL of tTA::miR132 mice, relative to control littermates that only express tTA (Fig. 4A-C). Western analysis of hippocampal lysates, showed a parallel attenuation of MeCP2 expression in tTA::miR132 versus tTA transgenic animals (Fig. 4D and E). These data support the aforementioned studies and thus, raise the possibility that transgenic miR132 could affect cognitive function.

### Overexpression of miR132 impairs novel object recognition memory

**Figure 5:**
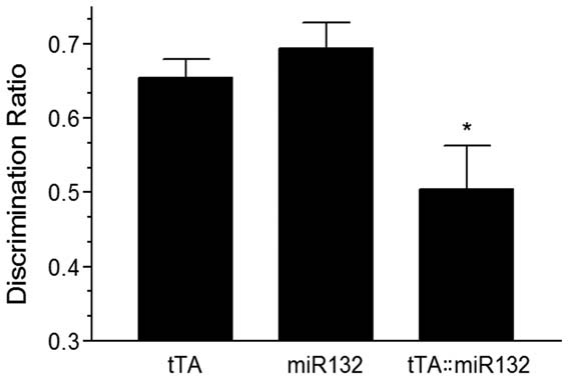
Decreased memory capability in mice over expressing miR132. Memory in tTA::miR132 bitransgenic mice was measured by novel object recognition. In contrast to monotransgenic littermates (tTA and miR132), which spent more time exploring novel objects than familiar ones, tTA::miR132 mice showed no significant capacity for object discrimination. Data are presented as mean discrimination ratio ±SEM, ANOVA F(2,15)  = 7.351, *p<.006, n = 6 for each group.

To elucidate the behavioral ramifications of transgenic miR132, we examined the recall memory of tTA::miR132 mice using a novel object recognition task. After two days of fifteen-min habituation trials in the testing field, mice were allowed to explore two identical objects for ten min and were then returned to their home cages. After a delay period of 30 min, one familiar object was replaced with a similar but novel object and the mice were allowed another five min of exploration time during which their interaction time with each object was measured. As expected in animals with a fully intact recall memory, monotransganic littermate control mice (i.e., tTA mice lacking the responder gene, and miR132 transgenic mice lacking the driver gene) spent more time with the novel object than the familiar one. In contrast however, tTA::miR132 bitransgenic mice showed no discrimination between the novel and familiar objects (Fig. 5). These data reveal a profound cognitive deficit resulting from dysregulation/overexpression of miR132.

## Discussion

Here, we explored the role of miR132 *in vivo* using a bitransgenic tTA::miR132 mouse model, in which miR132 was over expressed in excitatory neurons throughout the forebrain. Using a thy1-GFP morphological marker, we demonstrated that in CA1 pyramidal neurons, transgenic miR132 induces an increase in dendrite spine density. This finding is consistent with recent work using cell culture and brain slice-based methodologies, which showed that miR132 increased spinogenesis [13]. Further, the work of Impey et al. [13] showed that miR132 regulates synapse formation and function, thus raising the possibility that over-expression of miR132 affects synaptic communication *in vivo*. Future studies will examine this question in detail.

To test the functionality of transgenic miR132 at a molecular level, we examined the expression of MeCP2. This examination was based, in part, on recent work showing that the brain-enriched ‘long-form’ 3′ UTR of MeCP2 contains a phylogenetically conserved miR132 binding site, and that miR132 transfection of cortical neurons leads to a marked attenuation of MeCP2 expression [18]. Our data are consistent with these findings: transgenic miR132 led to a significant reduction in MeCP2 expression in the hippocampus.

Beyond simply validating the function of transgenic miR132, the data presented here, showing a down-regulation of MeCP2, has potentially significant ramifications for cognitive performance. MeCP2 is a multifunction protein that has been implicated in both the negative regulation of gene expression and RNA splicing [27]. As noted above, dysregulation of MeCP2 is associated with neuronal developmental abnormalities in Rett Syndrome [19]–[21]. Interestingly, in both Rett syndrome and in MeCP2-deficient mice, forebrain neurons exhibit a reduction in spine density [28]. This effect is somewhat inconsistent with the increase in spine density observed in the miR132 mice. One potential explanation for this discord is that miR132 targets a large number of mRNA species, some of which, such as *p250 GAP*, have been shown to *increase* neuronal complexity [29], [12]. Thus, the morphological phenotype observed here is the likely result of a complex interplay of loss- and gain-of-function physiological effects. Nevertheless, our data showing that miR132 markedly affects MeCP2 expression *in vivo*, provide a strong rationale for examining whether dysregulation of miR132 could be contributing to Rett Syndrome and other MeCP2-related disorders.

The effects of transgenic miR132 on both spine density and MeCP2 expression raised the possibility that miR132 influences cognitive performance. Indeed, we found that tTA::miR132 mice performed poorly on a hippocampal-dependent novel object recognition task, which is designed to test the integrity of recognition memory [24], [30]. This observation is of particular interest, given that the expression of endogenous miR132 is under the control of CREB [10], a transcription factor that plays a key role in regulating activity-dependent neuronal plasticity [31]. Interestingly, for optimal cognitive performance CREB-dependent transcription must be maintained within a limited range; hence, excessive CREB-mediated gene expression has been shown to interfere with hippocampal-dependent learning and memory [32]. This observation is consistent with our data on transgenic miR132 over-expression, and raises the interesting prospect that a more moderate increase in transgenic miR132 could reveal a facilitatory role for miR132 in learning and memory. Future studies in which transgenic expression of miR132 is carefully titered with doxycycline will test this possibility.

In conclusion, the findings reported here, coupled with prior work, indicate that miR132 is part of an activity-dependent gene expression program that underlies neuronal plasticity. Further work examining miR132 functionality in both health and disease in merited.
